# Identifying the key genes regulating mesenchymal stem cells chondrogenic differentiation: an in vitro study

**DOI:** 10.1186/s12891-022-05958-7

**Published:** 2022-11-15

**Authors:** Tongzhou Liang, Pengfei Li, Anjing Liang, Yuanxin Zhu, Xianjian Qiu, Jincheng Qiu, Yan Peng, Dongsheng Huang, Wenjie Gao, Bo Gao

**Affiliations:** 1 Department of Orthopedics, Sun Yat-Sen Memorial Hospital of Sun Yat-Sen University, Guangzhou, 510120 Guangdong China; 2 Department of Orthopaedics & Traumatology, Faculty of Medicine, Musculoskeletal Research Laboratory, The Chinese University of Hong Kong, Hong Kong SAR, China

**Keywords:** Mesenchymal Stem Cells, Chondrogenesis, Transcriptome Analysis, Transcriptional Factors

## Abstract

**Background:**

Mesenchymal stem cells (MSCs) possess the potential to differentiate into chondrocytes, which makes them an ideal source for healing cartilage defects. Here, we seek to identify the essential genes participating in MSCs chondrogenesis.

**Methods:**

Human MSCs were induced for chondrogenesis for 7, 14, and 21 days using a high-density micromass culture system, and RNA was extracted for RNA-seq.

**Results:**

A total of 6247 differentially expressed genes (DEGs) were identified on day 7, and 85 DEGs were identified on day 14. However, no significant DEGs was identified on day 21. The top 30 DEGs at day 7, including COL9A3, COL10A1, and CILP2, are closely related to extracellular matrix organization. While the top 30 DEGs at day 14 revealed that inflammation-related genes were enriched, including CXCL8, TLR2, and CCL20. We also conducted protein–protein interaction (PPI) networks analysis using the search tool for the retrieval of interacting genes (STRING) database and identified key hub genes, including CXCL8, TLR2, CCL20, and MMP3. The transcriptional factors were also analyzed, identifying the top 5 TFs: LEF1, FOXO1, RORA, BHLHE41, and SOX5. We demonstrated one particular TF, RORA, in promoting early MSCs chondrogenesis.

**Conclusions:**

Taken together, our results suggested that these DEGs may have a complex effect on MSCs chondrogenesis both synergistically and solitarily.

## Background

Cartilage tissues are responsible for attenuating force and providing lubrication for joint movement. Due to aging, sports injuries, or repeated use, cartilage defect is a major challenge in orthopedic surgery. Articular cartilage has limited regenerative ability, and cartilage injury can progress to osteoarthritis if poorly treated [1]. Stem cells combined with scaffolds have been used clinically as cartilage defect therapy [2]. The crucial process for these cell-based scaffolds to heal the cartilage defect requires cartilage differentiation (or chondrogenesis), but the regulatory factors involved are still not fully understood.

During cartilage development, the mesenchymal stem cells are recruited and undergo mesenchymal condensation and chondrogenic differentiation [3, 4]. Chondrogenesis is a tightly regulated process in which mesenchymal progenitors differentiate into mature chondrocytes and secrete extracellular matrix (ECM) components. The chondrocytes in the articular cartilage maintain the resting state and constitute hyaline cartilage. In pathologic conditions such as osteoarthritis's early stage, the chondrocytes undergo hypertrophic differentiation [5]. During chondrocyte hypertrophy, the ECM secreted by chondrocytes was changed from type II to type X collagen, leading to osteoarthritis progression [6]. However, the gene expression profile during the transition from resting to hypertrophic chondrocyte remained unclear.

Adult mesenchymal stem cells (MSCs) are pluripotent stem cells and can differentiate into multiple cell types, including osteocytes, chondrocytes, tenocytes, and adipocytes [6, 7]. Besides, MSCs exhibit other exciting properties, such as anti-inflammation, anti-senescence, and anti-allergic [8, 9]. Because of these properties and their simplicity of obtaining, MSCs injection has been used as a treatment for multiple clinical conditions [10]. Previous studies have shown that multiple genes and pathways are crucial to the chondrogenesis of MSCs, such as SOX9 and BMP signaling pathways [11]. However, despite these well-established genes and pathways that regulate chondrogenesis, emerging evidence has shown that chondrogenesis is regulated by a complex network of other participants, such as non-coding RNA and growth factors [12]. Therefore, further studies are still needed to analyze the dynamic change during MSC chondrogenesis.

To investigate the vital genes in regulating MSCs chondrogenesis, it is important to characterize the gene expression pattern during chondrogenesis. In this study, MSCs chondrogenesis was induced by a high-density micromass culture system. We then performed RNA-seq and subsequent analysis at different time points during MSCs chondrogenic differentiation. We hope this study may enhance our understanding of the gene profile during chondrogenesis. More importantly, identifying the key transcriptional factors that initiate and maintain MSCs chondrogenesis in vitro may enhance our comprehension on the mechanism of induced chondrogenesis in vivo.

## Methods

### MSC isolation and culture

The Ethical Committee of Sun Yat-sen University approved this study. Every participant enrolled in the study signed a formal consent form for collecting MSCs. The MSCs from three male participants with an average age of 37.6 ± 4.2 years were used for chondrogenic differentiation. Briefly, the bone marrow aspirate was collected from the posterior iliac crest. Bone marrow aspirate was collected using a 14-gauge needle connected to a 20-mL syringe. Approximately 2 mL of bone marrow was collected from each puncture in the posterior iliac crest. The bone marrow aspirate was immediately transferred to a vacutainer tube containing the anticoagulant. MSCs were separated from bone marrow aspirates as described previously [13, 14]. Briefly, the bone marrow aspirates were diluted with sterile phosphate buffer saline (PBS) and then fractionated with density gradient by 500 g for 30 min. The mononuclear cells layer was carefully collected and resuspended in low-glucose Dulbecco’s modified Eagle medium (DMEM; Gibco, Waltham, MA, USA). The MSCs were incubated at 37 ℃ incubators at 5% CO2. The medium was replaced every 3 days and was digested with 0.25% trypsin (Gibco, Waltham, MA, USA) for passage.

### Chondrogenic differentiation

As previously described, the chondrogenic differentiation of MSCs was achieved using a high-density micromass culture system [14, 15]. Only MSCs from passages 3–5 were subjected to chondrogenic differentiation. The procedure of chondrogenic differentiation was described as follows: MSCs were separated with 0.25% trypsin and washed with PBS. MSCs were suspended at a cell number of 2×10^7^ Cells/ml in Mesenchymal Stem Cell Chondrogenic Differentiation Medium (MCDM, ScienCell) supplemented with 10ug/ml TGF-β3 (PEPROTECH, USA, Cat No: 100-36E). Each MSCs suspension droplet (12.5 μl) was carefully placed on a 24-well plate. The droplets were then placed in the 37 ℃ incubators for 2 h to evaporate liquid and form aggregate. Then 500 μl chondrogenic medium was added to each well. The medium was replaced every 3 days, and the pellets were harvested after 7, 14, and 21 days of induction. The CHO group was chondrocytes isolated from an age-paired patient who underwent total knee arthroplasty (TKA) surgery. The relatively intact part of the knee cartilage was dissected, the chondrocytes were digested, and total RNA was extracted.

### RNA extraction and RNA-seq

Total RNA was extracted from the cartilage pellets with TRIzol reagent (Invitrogen). A total amount of 1 µg RNA per sample was used as input material for the RNA sample preparations. Cartilage pellets were sent to Novogene (Beijing, China) for RNA-seq analysis. Briefly, RNA integrity number (RIN) was evaluated by Agilent 2100 Bioanalyzer. The sequencing libraries were generated and sequenced by an Illumina HiSeq X Ten sequencer (Illumina).

### Data processing and the differentially expressed genes (DEGs) analysis

We calculated the RPKM (reads per thousand base exons per million mapped reads) value to evaluate gene expression. The R software package FlashClust was used to cluster the genes expressed in different samples. The DESeq package was used for differential gene expression analysis at different time-point. The false discovery rate (FDR) was controlled by adjusting the *p*-value using the Benjamin-Hochberg algorithm. The significant DEGs were identified as log2FoldChange > 1 or < -1, *P* < 0.05. The R package ggPlot2 and pheatmap were used to visualize the DEGs.

### Protein–protein interaction (PPI) network analysis

The DEGs in the form of gene entrez was uploaded to the STRING database for PPI network analysis (https://www.string-db.org/). Briefly, the selection criteria were as follows: organism: Homo sapiens; network: full network; interaction score: high confidence. Each hub gene is clustered by kmeans clustering. The networks were further analyzed by Cytoscape version 3.8.2 (http://cytoscape.org/) to identify hub genes and their relationships. The inserted app cytoHubba and MCODE was used to assist the analysis.

### Statistical analysis

The numeric data were presented as the mean ± SD of three replicates. SPSS 23.0 was used to perform the statistical analysis. GraphPad Prism 8.0 software was used to visualize the results. Differences between the two grouped data were analyzed with the student's t-test.

## Results

### Identification of DEGs during MSCs chondrogenesis

**Fig. 1:**
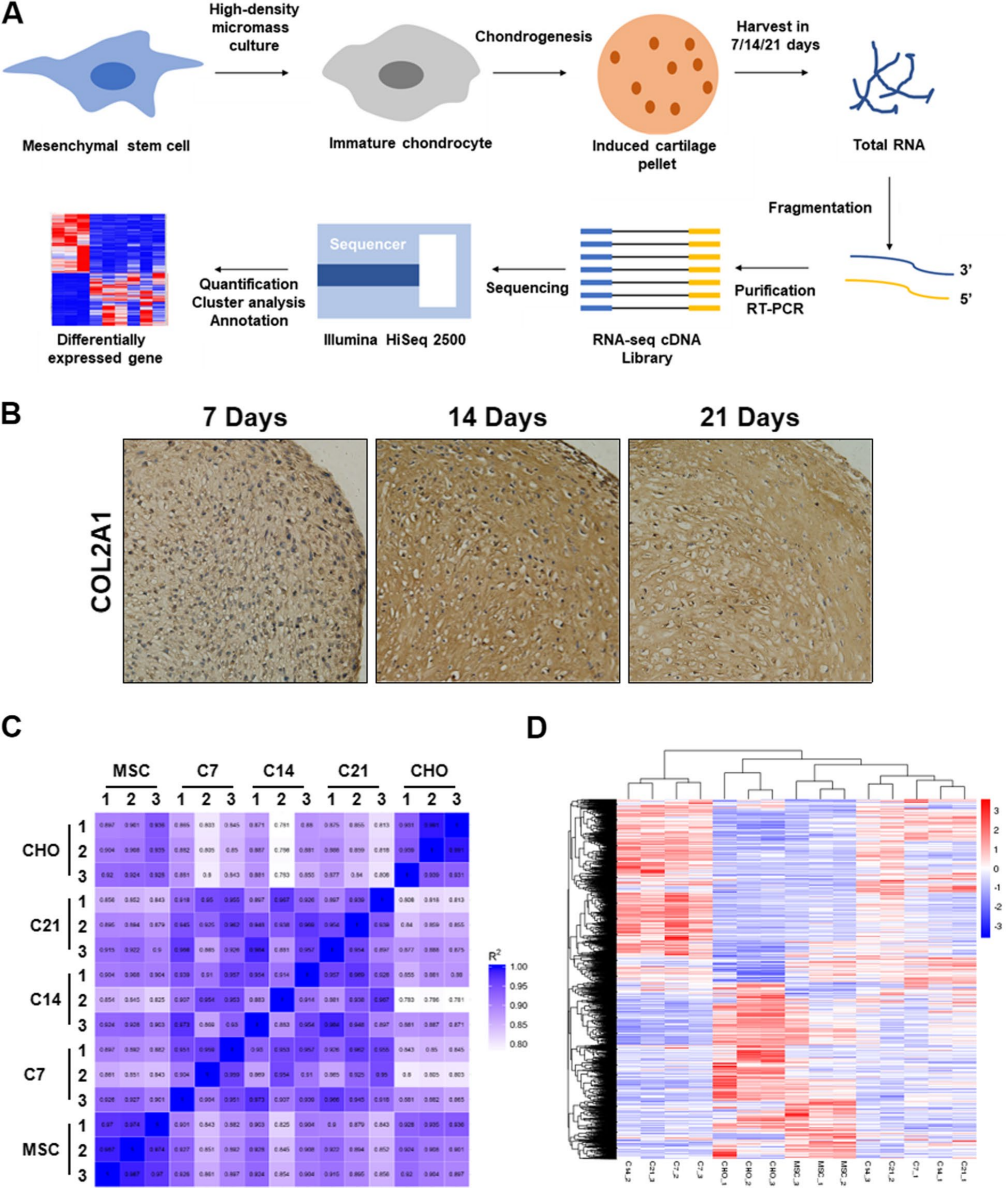
Study design and heatmap showing all DEGs. **A** The expreimental procedure of this study. **B** The expression of chondrogenic marker COL2A1 in chondrocyte pellet after induced for 7, 14, and 21 days were assessed by IHC. **C** The correlation coefficient between different biological replicates and indicated time points. MSCs = undiferentiated MSCs, C7 = induced chondrogenesis for 7 days, C14 = induced chondrogenesis for 14 days, C21 = induced chondrogenesis for 21 days, CHO = mature chondrocytes. **D** Heatmap showing all DEGs. The expression values are |log2 fold changes|≥ 1 and P ≤ 0.05

The schematic illustration of this experiment was shown in Fig. 1A. We performed immunohistochemistry(IHC) staining to validate the expression of the chondrogenic marker COL2A1. The successful induction of MSCs chondrogenesis was confirmed (Fig. 1B). The samples were then collected and subjected to RNA sequencing, described in the method section. Pearson’s correlation between different groups was presented as R2 value. The in-group difference was more negligible than the inter-group (Fig. 1C). As shown in Fig. 1D, there was a significant difference in the gene expression profile between different time points.

**Fig. 2:**
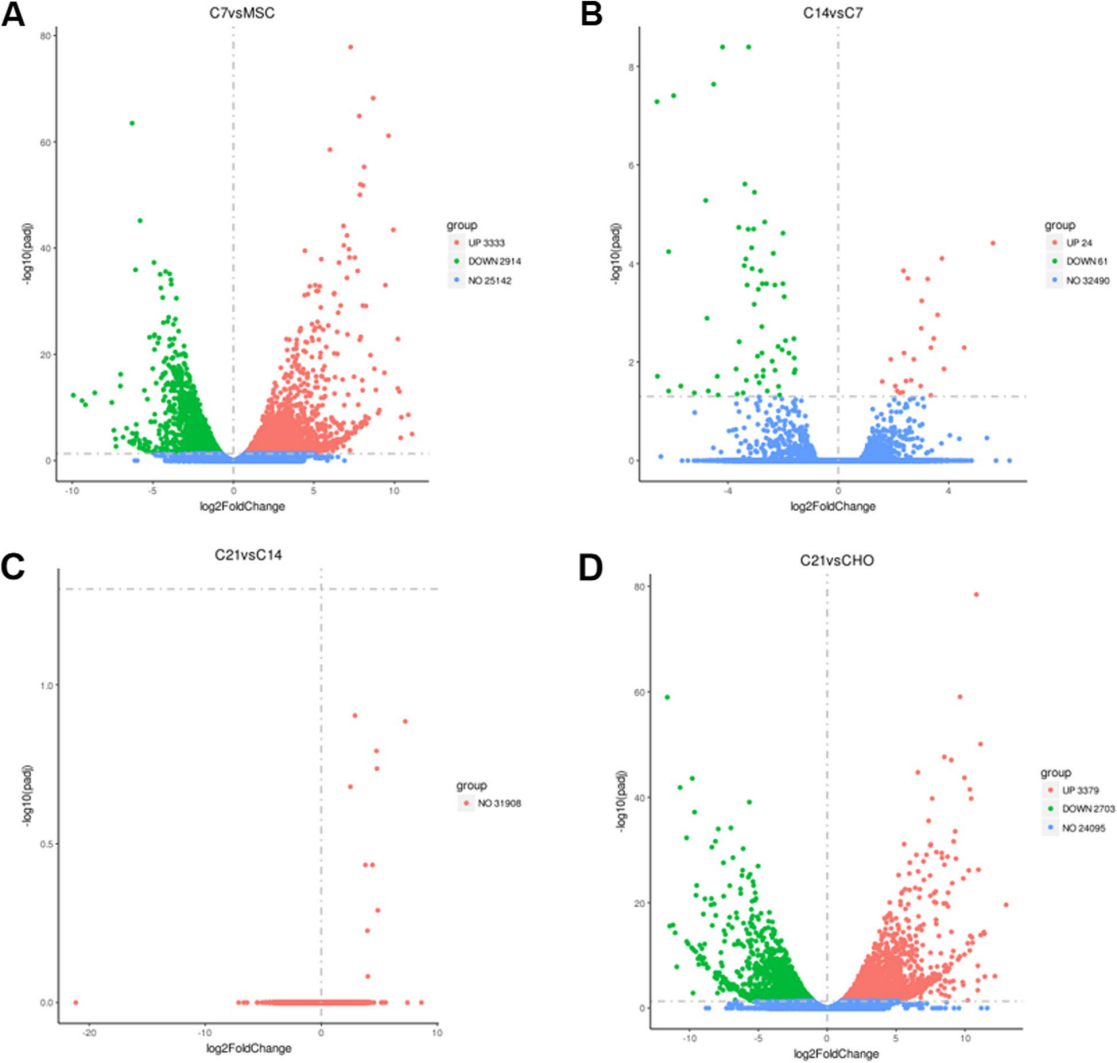
Volcano plot showing DEGs. **A** DEGs between MSC and C7 group. **B** DEGs between C7 and C14 group. **C** DEGs between C14 and C21 group. **D** DEGs between C21 and CHO group

The pellet harvested on day 7 was identified 6247 DEGs compared to undifferentiated MSCs. Among the 6247 DEGs, 3333 genes were up-regulated, and 2914 genes were down-regulated (Fig. 2A). Compared to the pellet harvested on day 7, a total of 85 DEGs was identified in the pellet harvested on day 14. Among the 85 DEGs, 24 were up-regulated, and 61 were down-regulated (Fig. 2B). The top 30 DEGs at 7 days after induced chondrogenesis was shown in Table 1, and the top 30 DEGs at 14 days compared to 7 days were shown in Table 2. However, there were no statistically significant DEGs between day 21 and day 14 pellet, suggesting the induced chondrogenesis was halted (Fig. 2C & D).

**Table 1:** Top 30 markedly differentially expressed genes between MSC and 7 days after induced chondrogenesis

Gene	LogFC	*P* Value	Padj	Gene	LogFC	*P* Value	Padj
CILP2	7.279121	6.61E-83	1.47E-78	CCBE1	-5.79104	3.53E-49	7.15E-46
H19	8.676741	5.54E-73	6.16E-69	LEF1	6.834446	3.94E-48	7.30E-45
APOD	7.811963	1.91E-69	1.42E-65	ALOX15B	9.931582	2.15E-47	3.67E-44
RGS4	-6.28539	5.82E-68	3.24E-64	COL9A2	7.051985	2.68E-46	4.26E-43
OMD	9.629494	1.63E-65	7.25E-62	FGFR3	6.85564	2.11E-44	3.13E-41
MGP	5.995104	8.14E-63	3.02E-59	COL10A1	7.179266	1.13E-43	1.58E-40
NEBL	8.117441	1.74E-59	5.55E-56	MT1X	4.442558	2.43E-43	3.19E-40
WDR86	7.881255	3.57E-56	9.93E-53	KRT16	7.202662	4.63E-42	5.73E-39
COL9A3	8.053868	6.59E-56	1.63E-52	C5AR2	7.539788	5.34E-42	6.25E-39
CAPN6	7.859288	4.26E-54	9.48E-51	PTGES	5.458614	1.13E-41	1.26E-38

**Table 2:** Top 30 markedly differentially expressed genes between 7 and 14 days after induced chondrogenesis

Gene	LogFC	*P* Value	Padj	Gene	LogFC	*P* Value	Padj
PTGS1	-3.24743	2.37E-13	4.06E-09	TFPI2	-3.06721	1.22E-08	2.00E-05
AREG	-4.1923	4.50E-13	4.06E-09	RPSAP52	-3.26671	1.34E-08	2.02E-05
HSD11B1	-4.51604	3.84E-12	2.31E-08	SOD2	-2.00261	1.75E-08	2.42E-05
CXCL8	-5.96364	8.74E-12	3.94E-08	COL26A1	5.604523	3.01E-08	3.87E-05
IL24	-6.56486	1.44E-11	5.21E-08	TREM1	-3.14162	3.99E-08	4.80E-05
SFRP1	-3.38346	8.13E-10	2.44E-06	MROH9	-6.14284	5.10E-08	5.74E-05
GALNT15	-3.03558	1.39E-09	3.58E-06	AKR1C2	-2.35332	5.88E-08	6.24E-05
CCL20	-4.79915	2.34E-09	5.26E-06	F13A1	3.753615	7.91E-08	7.92E-05
DUSP4	-2.66425	7.20E-09	1.44E-05	TLR2	-3.34797	8.56E-08	8.12E-05
DNER	-3.60495	1.03E-08	1.86E-05	FOXQ1	-3.40526	1.23E-07	0.000111

### Functional GO terms and pathway enrichment analysis

**Fig. 3:**
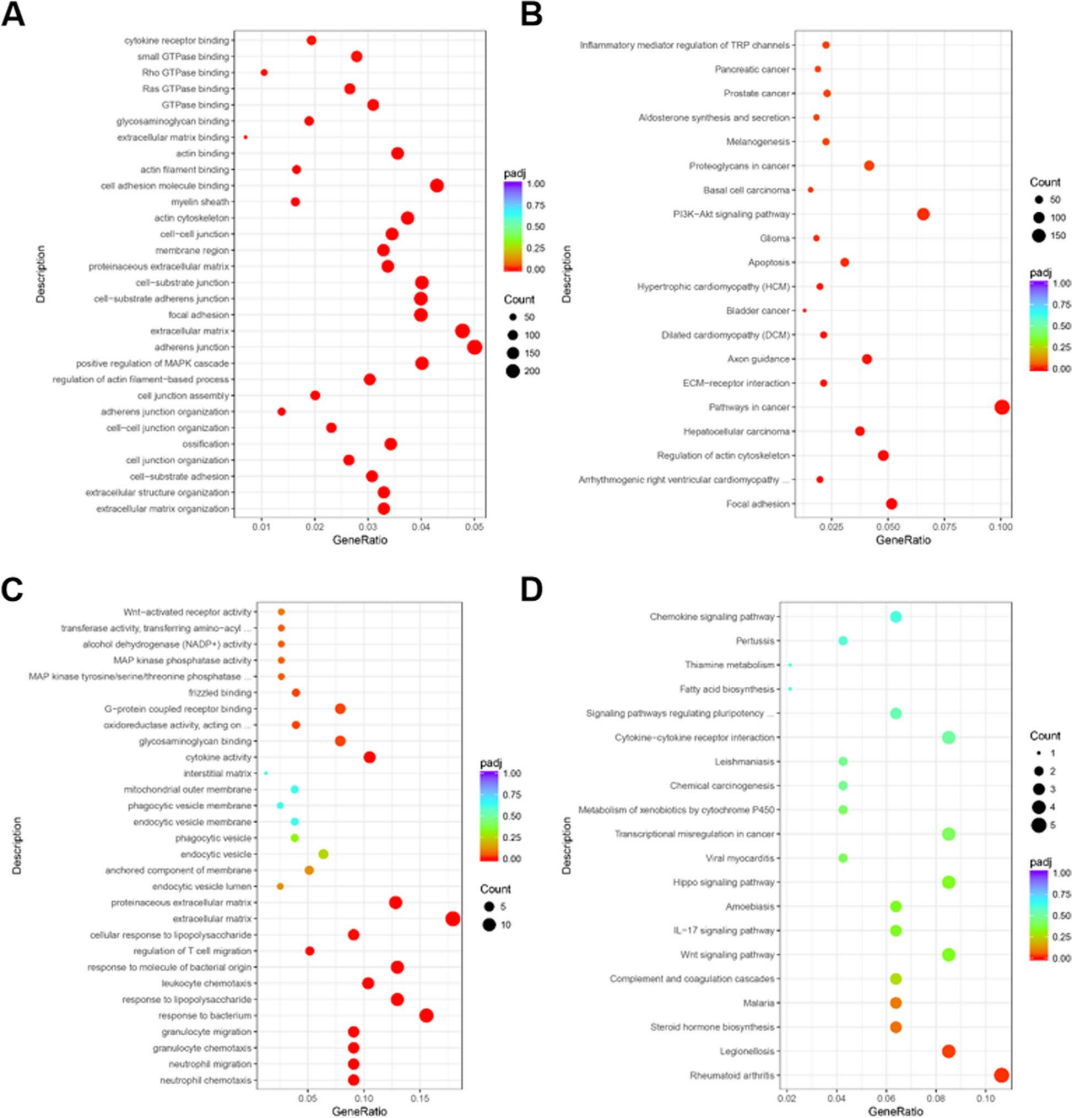
GO and KEGG analysis of DEGs in day 7 and day 14. **A** & **B** The GO and KEGG analysis of the 6247 DEGs between MSC and C7 group. **C** & **D** The GO and KEGG analysis of the 85 DEGs between C7 and C14 group

The GO functional annotation analysis of the 6247 DEGs at day 7 revealed that extracellular matrix organization (*P* = 9.13E-11), extracellular structure organization (*P* = 1.22E-10), cell-substrate adhesion (*P* = 2.65E-09), cell junction organization (*P* = 5.54E-09), and ossification (*P* = 3.89E-08) were the top 5 altered biological processes (Fig. 3A). The KEGG functional analysis of these DEGs at day 7 revealed that focal adhesion (*P* = 9.88E-09), arrhythmogenic right ventricular cardiomyopathy (*P* = 7.96E-06), regulation of actin cytoskeleton (*P* = 9.07E-06), hepatocellular carcinoma (*P* = 3.87E-05) and pathways in cancer (*P* = 4.74E-05) were the top 5 altered pathways (Fig. 3B).

The GO functional annotation analysis of 85 DEGs on day 14 was also analyzed. The top 5 terms of biological processes included neutrophil chemotaxis (*P* = 8.11E-08), neutrophil migration (*P* = 1.55E-07), granulocyte chemotaxis (*P* = 4.12E-07), granulocyte migration (*P* = 8.41E-07), and response to bacterium (*P* = 1.47E-06) (Fig. 3C). These biological processes are related to common genes, including CXCL8, CCL20, TREM1, RAC2, SAA1, RIPOR2, and ITGB2. The KEGG functional analysis of these DEGs at day 14 identified two pathways of significance: rheumatoid arthritis (*P* = 1.38E-04) and legionellosis (*P* = 4.35E-04) (Fig. 3D).

### PPI network analysis

**Fig. 4:**
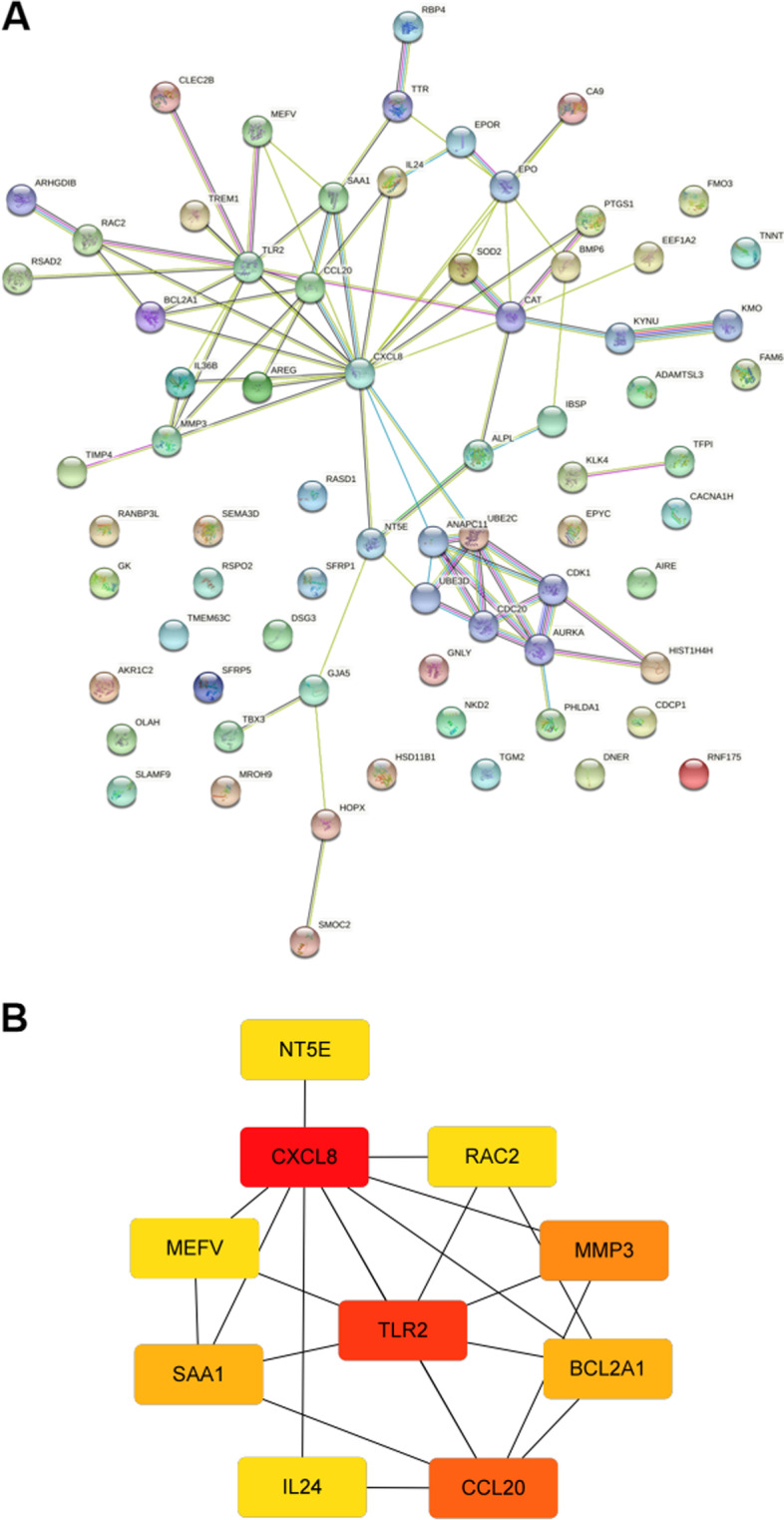
PPI network of co-DEGs between day 7 and day 14. **A** PPI network of 65 co-DEGs in C7 and C14 group. **B** The top 10 hub nodes of the PPI network as analyzed by cytoHubba

In order to identify the key DEGs during the whole process of chondrogenesis, the common DEGs in the 7 days chondrogenesis group and 14 days chondrogenesis group were identified and selected. A total of 65 DEGs were identified and subjected to STRING for PPI analysis. The results of the PPI network are shown (Fig. 4A). To eliminate unrelated genes and to identify hub node genes in the process, the results were further analyzed with Cytoscape and cytoHubba. The top 10 hub nodes were identified, including CXCL8, TLR2, CCL20, MMP3, BCL2A1, SAA1, NT5E, RAC2, IL24, and MEFV (Fig. 4B).

### Identification of key transcription factors during chondrogenesis

Transcriptional factors (TFs) are crucial in initiating the chondrogenic process. Therefore, we screened the top 5 markedly differentially expressed TFs on day 7 (Table 3). Top 5 differentially expressed TFs includes LEF1 (*P* = 3.94E-48), FOXO1 (*P* = 6.59E-26), RORA (*P* = 4.46E-24), BHLHE41 (*P* = 6.68E-24), SOX5 (*P* = 3.45E-20). The TF differentially expressed on day 14 was also analyzed. FOXQ1 (*P* = 1.23E-07) and TBX3 (*P* = 1.77E-05) were identified as markedly differentially expressed TFs on day 14 (Table 4).

**Table 3:**
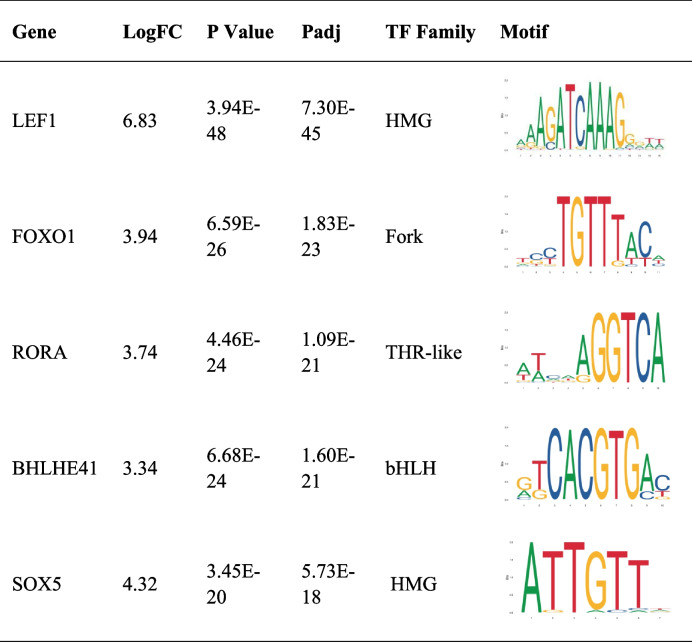
Top 5 markedly differentially expressed transcriptional factors 7 days after induced chondrogenesis

**Table 4:**
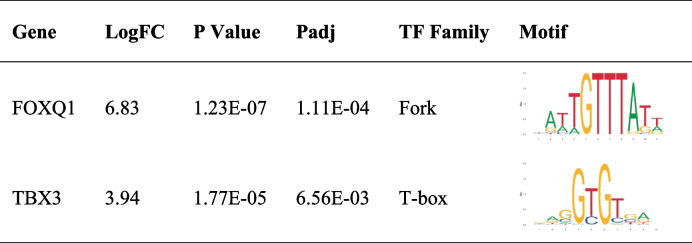
Markedly differentially expressed transcriptional factors 14 days after induced chondrogenesis

### Validation on transcriptional factors involved in chondrogenesis

**Fig. 5:**
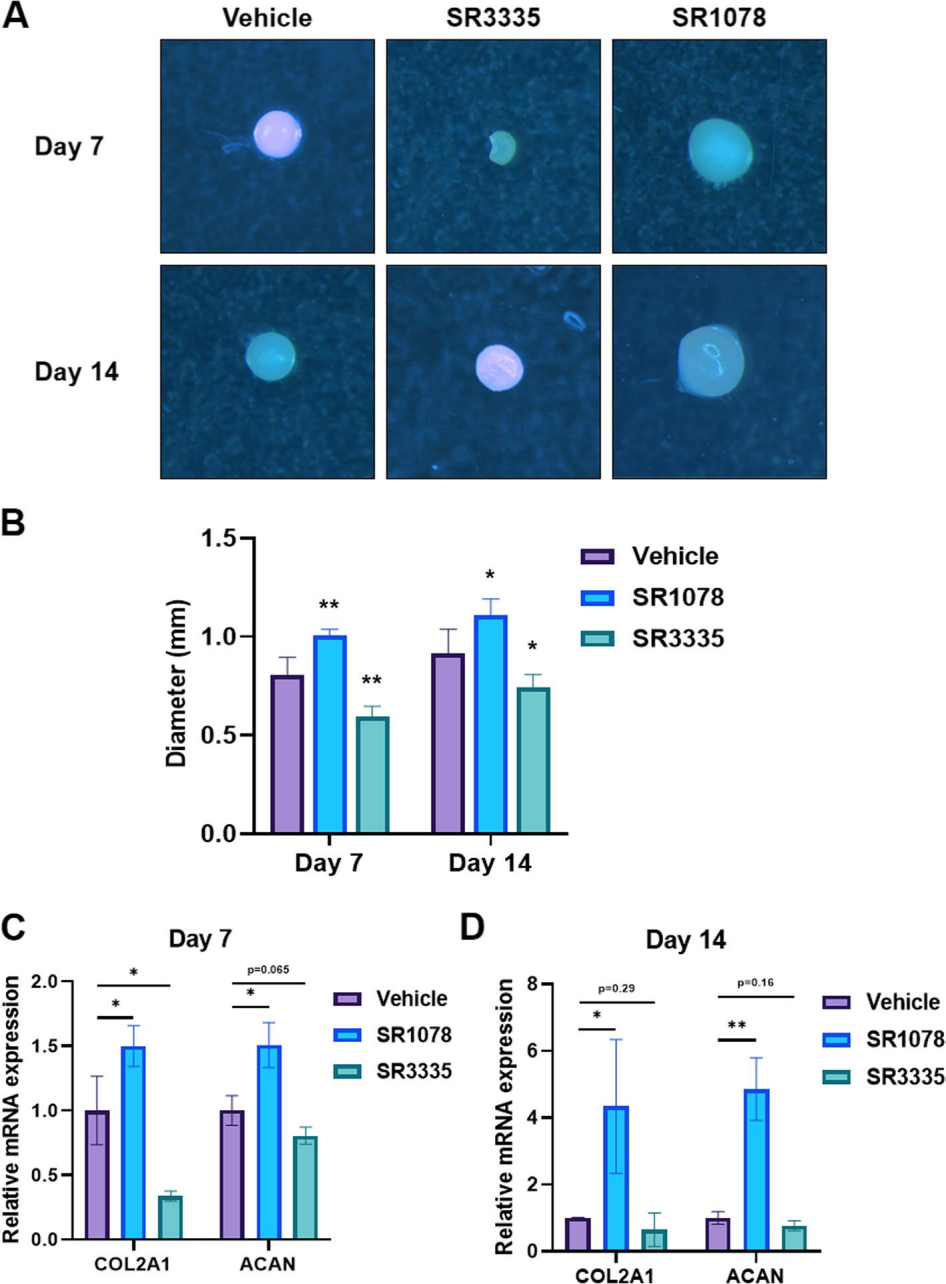
RORA promotes MSCs chondrogenesis. **A** Cartilage pellets collected 7 and 14 days after inducing MSCs chondrogenesis. Indicated treatment was added to the culture media. The cartilage pellets were observed with stereoscopic microscope. **B** The diameter (mm) of cartilage pellet after 7 and 14 days of induced chondrogenesis, 6 pellets were used for measurement in each group. **C** RT-qPCR analysis was conducted to assess the mRNA expression of COL2A1 and ACAN in chondrocyte pellet with the addition of indicated treatments for 7 days. **D** RT-qPCR analysis was conducted to assess the mRNA expression of COL2A1 and ACAN in chondrocyte pellet with the addition of indicated treatments for 14 days.* *p* ≤ 0.05 and ** *p* ≤ 0.01 compared to the vehicle treated group

During MSCs chondrogenesis, the initial stage (before day 7) was regarded as the most critical stage in determining MSCs' fate [16]. Therefore, we focused on the TFs that are differentially expressed on day 7. The role of LEF1, FOXO1, and SOX5 in MSCs chondrogenesis has been identified [17–19]. Because our previous studies show that melatonin promotes chondrogenic differentiation [15, 20], we are particularly interested in RORA, the nuclear receptor for melatonin. Treatment throughout the induction process with an antagonist for RORA SR3335 (1 μM) attenuated the effect of induced chondrogenesis. While continued treatment with 1 μM SR1078, an agonist for RORA, promoted chondrogenesis (Fig. 5A & B). The expression of chondrogenesis marker genes COL2A1 and ACAN were upregulated upon SR1078 treatment after 7 and 14 days of induction (Fig. 5C). When treated with SR3335 for 7 days, the expression level of COL2A1 was suppressed, but no significant difference between the vehicle and SR3335 in 14 days (Fig. 5D).

## Discussion

In this study, we identified the transcriptional profile during chondrogenesis of human MSCs. Recent studies have demonstrated that MSCs are promising therapies for treating musculoskeletal disorders [21, 22]. Inducing MSCs chondrogenesis is an especially promising strategy to treat cartilage defects because of its low immunogenicity and capability to repair cartilage defects in situ. However, the mechanism underlying different stages during chondrogenesis remained relatively unknown. Therefore, identifying key genes and pathways may provide new insight into optimizing conditions of chondrogenesis both in vitro and in vivo.

Cartilage regeneration is a promising disease-modifying OA therapy [23]. The cartilage regeneration process involves stem cell differentiation and ECM synthesis. However, the therapeutic application of induced chondrogenesis is limited, partially due to the declined differentiation capacity of MSCs. Osteoarthritis and cartilage defects create an inflammatory microenvironment, which makes MSCs aberrantly differentiate to the fibroblast phenotype [24]. Therefore, understanding the key regulatory factors can contribute to optimizing the chondrogenic method and microenvironment. For example, activating the pro-inflammatory IL-6/STAT-3 pathway promotes chondrogenesis, revealing that inflammation may play a dual role in regulating chondrogenesis [25]. However, overproduction and accumulation of the pro-inflammatory factor IL-1β hamper the chondrogenic differentiation of MSCs [14].In this study, we found that neutrophil-related cytokines are significantly upregulated during chondrogenesis. Similar to our findings, another study found that immune response-related DEGs, including CXCL8 and CCL20, are upregulated during chondrogenesis [26].

The PPI analysis suggested some important hub genes in the network, as shown in Fig. 3B, including CXCL8, TLR2, CCL20, MMP3, BCL2A1, SAA1, RAC2, and IL24. These genes are closely related to regulating inflammation and response to inflammatory cytokines. Interestingly, top DEGs and hub genes reveal some common genes, including CXCL8, CCL20, SAA1, and RAC2. CXCL8, a member of the CXC chemokine family, encodes the interleukin-8 (IL-8) protein. The role of IL-8 in chondrogenesis remained controversial. IL-8 stimulates chondrocyte growth and differentiation and may contribute to cartilage defect repair [27, 28]. In contrast, other studies suggested that IL-8 may promote cartilage destruction during the pathogenesis of osteoarthritis [29, 30]. Therefore, the role of CXCL8 in chondrogenesis remains unclear and requires further exploration. As for TLR2 (encoding Toll-like receptor 2), a study by Lee et al*.* demonstrated that TLR2 promotes chondrogenic differentiation and consequent calcification in vascular smooth muscle cells [31]. Moreover, studies found that TLR2 is expressed in MSCs, and treatment with TLR2 agonists promotes chondrogenesis [32]. Interestingly, stimulation with TLR2 agonists led to increased production of IL-8 in MSCs in a dose-dependent manner [33]. Therefore, TLR2 and CXCL8 may represent a new pathway in regulating chondrogenesis and are worth further exploration.

We also identified several critical TFs that participated in chondrogenesis, including LEF1, FOXO1, RORA, BHLHE41, and SOX5. LEF1 forms a transcriptional complex with β-catenin and acts as an important downstream effector of the Wnt/β-catenin signaling pathway. A study showed that LEF1 cooperates with WNT5A and WNT11, directs MSCs chondrogenesis, and prevents chondrocytes from hypertrophic differentiation [17]. Besides, FOXO1 knockdown attenuated chondrogenesis by regulating SOX9 expression [18]. The role of RORA in MSCs chondrogenesis has not been investigated. We found that RORA pharmacological inhibition attenuated MSCs chondrogenesis while activating RORA promoted chondrogenesis. Previous studies suggested that RORA plays a contributory role in the pathogenesis of osteoarthritis by regulating cholesterol metabolism and IL6/STAT3 pathway [34, 35]. RORA also regulates chondrocytes hypertrophy in tibia organ cultures isolated from E15.5 mice [36]. These results suggested that different from LEF1, RORA may promote chondrogenesis and chondrocyte hypertrophy simutaneously.

In conclusion, this study provided insight into the gene expression profile during MSCs chondrogenesis. The hub genes and key transcriptional factors were identified, which may assist the research for cartilage regeneration and the development of osteoarthritis in future studies.

## Data Availability

The datasets generated and/or analysed during the current study are available in the GEO repository, accession number GSE210984 (https://www.ncbi.nlm.nih.gov/geo/query/acc.cgi?acc=GSE210984).

## Abbreviation

MSCs: Mesenchymal stem cells
TF: Transcription factor
FC: Fold change
DEGs: Differentially expressed genes
KEGG: Kyoto Encyclopedia of Genes and Genomes
GO: Gene Ontology
PPI: Protein–protein interaction
RIN: RNA integrity number
